# Supplementary material for: Exposure to Oxidized Multi-Walled CNTs Can Lead to Oxidative Stress in the Asian Freshwater Clam Corbicula fluminea (Müller, 1774)
Source: Int J Mol Sci. 2023 Nov 9;24(22):16122. doi: 10.3390/ijms242216122 (PMC10671163; doi:10.3390/ijms242216122)
Supplement: Supplementary file 1 [file ijms-24-16122-s001.zip › ijms-2699321-supplementary.pdf]

International Journal of *Molecular Sciences*

MDPI

Table S1. Catalase activities measured in gills and in digestive glands of *C. fluminea* exposed to different concentrations of Ox-MWCNTs and exposure periods.

	**Catalase activity/nmol.min^{-1}.mg^{-1} total protein**							
	**7 days of exposure**				**14 days of exposure**			
	G		DG		G		DG [1]	
**mg Ox-MWCNTs. L^{-1}**	Mean	S. d.[1]	Mean	S. d.	Mean	S. d.	Mean	S. d.
0	0.19	0.13	0.58	0.33	0.05	0.03	0.33	0.10
0.1	1.02	0.21	1.96	0.60	0.49	0.15	1.75	0.62
0.2	0.29	0.11	1.43	0.93	0.29	0.17	1.17	0.21
0.5	0.44	0.33	0.97	0.80	0.20	0.02	0.61	0.16

[1]S.d.- standard deviation

Table S2. GST activities measured in gills and in digestive glands of *C. fluminea* exposed to different concentrations of Ox-MWCNTs and exposure periods.

	**GST activity/nmol.min^{-1}.mg^{-1} total protein**							
	**7 days of exposure**				**14 days of exposures**			
	G		DG		G		DG [1]	
**mg Ox-MWCNTs. L^{-1}**	Mean	S. d.[1]	Mean	S. d.	Mean	S. d.	Mean	S. d.
0	2.17	0.87	7.19	3.48	2.64	0.87	6.80	2.55
0.1	1.10	0.46	6.15	3.50	3.35	1.44	10.76	3.29
0.2	2.28	1.90	4.10	1.62	6.25	2.92	8.93	4.99
0.5	1.79	0.77	5.62	3.28	4.27	1.32	8.73	4.53

[1]S.d.- standard deviation

Table S3. MDA contents measured in gills and in digestive glands of *C. fluminea* exposed to different concentrations of Ox-MWCNTs and exposure periods.

	**MDA contents/nmol.min^{-1}.mg^{-1} total protein**							
	**7 days of exposure**				**14 days of exposure**			
	G		DG		G		DG [1]	
**mg Ox-MWCNTs. L^{-1}**	Mean	S. d.[1]	Mean	S. d.	Mean	S. d.	Mean	S. d.
0	0.59	0.44	0.90	0.74	1.90	0.72	1.55	0.55
0.1	1.13	1.01	0.40	0.31	0.40	0.37	1.59	1.55
0.2	0.86	0.67	0.35	0.29	0.25	0.26	0.32	0.50
0.5	0.53	0.48	0.58	0.43	0.93	0.46	0.66	0.63

[1]S.d.- standard deviation

Table S4. SOD activities measured in gills and in digestive glands of *C. fluminea* exposed to different concentrations of Ox-MWCNTs and exposure periods.

	**SOD/Units.mg^{-1} total protein**							
	**7 days of exposure**				**14 days of exposure**			
	G		DG		G		DG [1]	
**mg Ox-MWCNTs. L^{-1}**	Mean	S. d.[1]	Mean	S. d.	Mean	S. d.	Mean	S. d.
0	5.65	0.91	4.97	1.48	3.71	0.17	3.71	0.20
0.1	3.34	0.01	3.34	0.02	3.97	0.34	4.65	1.11
0.2	3.76	1.66	3.83	0.55	3.35	0.01	3.35	0.01
0.5	3.32	0.02	3.32	0.03	3.36	0.03	3.34	0.04

[1]S.d.- standard deviation

Table S5. Total ubiquitin measured in gills and in digestive glands of *C. fluminea* exposed to different concentrations of Ox-MWCNTs and exposure periods.

	**Total Ub/µg.mg^{-1} of total protein**							
	**7 days of exposure**				**14 days of exposure**			
	G		DG		G		DG [1]	
**mg Ox-MWCNTs. L^{-1}**	Mean	S. d.[1]	Mean	S. d.	Mean	S. d.	Mean	S. d.
0	0.013	0.002	0.009	0.001	0.008	0.001	0.006	0.001
0.1	0.014	0.006	0.009	0.001	0.013	0.002	0.008	0.002
0.2	0.007	0.002	0.005	0.002	0.012	0.004	0.005	0.002
0.5	0.013	0.003	0.007	0.004	0.007	0.004	0.005	0.002

[1]S.d.- standard deviation
